# Psychological factors associated with pain and function in adults with hallux valgus

**DOI:** 10.1002/jfa2.70030

**Published:** 2025-03-02

**Authors:** Abdel Kak, Mehak Batra, Bircan Erbas, Sean Sadler, Vivienne Chuter, Jeffery Jenkins, Haydar Ozcan, Damien Lafferty, Ozan Amir, Matthew Cotchett

**Affiliations:** ^1^ School of Health Sciences Western Sydney University Campbelltown New South Wales Australia; ^2^ School of Psychology and Public Health La Trobe University Melbourne Victoria Australia; ^3^ Discipline of Podiatry University of Newcastle Newcastle New South Wales Australia; ^4^ Sydney Foot Surgery Edgecliff New South Wales Australia; ^5^ Manly Foot Clinic North Manly New South Wales Australia; ^6^ Department of Physiotherapy, Podiatry, Prosthetics and Orthotics School of Allied Health Human Services and Sport La Trobe University Melbourne Victoria Australia

**Keywords:** anxiety, catastrophization, foot, kinesiophobia, pain, stress

## Abstract

**Introduction:**

Psychological factors are linked to pain and function in various musculoskeletal conditions, but their impact on hallux valgus is unclear. Health‐related quality of life declines with increasing severity of hallux valgus, affecting not only foot pain and physical function, but also general health, vitality and mental health. Previous studies have reported inconsistent associations between psychological factors, such as anxiety and depression, and surgical outcomes, which might relate to variability in measurement approaches. Understanding the associations between psychological factors, including anxiety, depression, pain catastrophizing and kinesiophobia, and hallux valgus‐related pain and function may inform more holistic pre‐operative care. Therefore, we aimed to assess these associations in adults with hallux valgus pre‐surgery.

**Methods:**

A pre‐operative cross‐sectional study was conducted with 41 adults scheduled for hallux valgus surgery. Participants completed questionnaires measuring continuous psychological variables: depression, anxiety and stress (Depression Anxiety Stress Scale‐21, a tool for general psychological distress), kinesiophobia (Tampa Scale for Kinesiophobia, which assesses fear of movement associated with pain) and pain catastrophizing (Pain Catastrophizing Scale, a tool used to evaluate maladaptive pain‐coping strategies). Continuous outcomes were evaluated using the Manchester–Oxford Foot Questionnaire for foot function, pain and social interaction. Multiple linear regressions explored the associations between these psychological factors and the outcomes.

**Results:**

When all exposure variables were considered simultaneously, pain catastrophizing emerged as a significant predictor of foot pain and foot function. A one‐unit increase in the pain catastrophizing score was associated with a 1.41‐point increase in foot pain (*β* = 1.41, 95% confidence intervals (CIs) 0.73–2.09 and *p* < 0.001) and a 1.83‐point increase in worse foot function (*β* = 1.83, 95% CI 1.12–2.54 and *p* < 0.001).

**Conclusion:**

Assessing pain catastrophising pre‐operatively is recommended for individuals with hallux valgus, although more structured education may be needed to support health professionals in assessing psychological factors. Future research should evaluate the longitudinal impact of pain catastrophizing on post‐operative outcomes and explore other contributing factors, such as comorbidities, lifestyle variables and sex differences, to refine screening and treatment strategies.

## 1 BACKGROUND

Hallux valgus is considered a chronic, progressive and degenerative condition [1] that is characterised by a medial prominence of the first metatarsal head, lateral deviation and displacement of the hallux at the first metatarsophalangeal joint (MTPJ) [2]. The condition can be accompanied by pain, deformity, muscle weakness, keratotic lesions, changes in the lesser toes and difficulties with footwear fitting [3]. The global estimated pooled prevalence of hallux valgus is 19% with a higher prevalence found in females (23.74%) compared to males (11.43%) [4]. The prevalence of hallux valgus is highest among individuals aged over 60 years (22.7%) compared to those younger than 20 years (11%) and adults aged 20–60 (12.22%) [4].

Hallux valgus has been found to have a negative impact on health‐related quality of life [5] and is linked to presence of foot pain [6], altered walking patterns [7], impaired balance [8] and increased falls in older adults [9]. Consequently, it is recognised as a major public health problem, with a high incidence and cost related to foot surgery [10].

The experience of pain is associated with biological, psychological and social factors [11]. Psychological factors, including depression, anxiety, stress, pain catastrophizing and kinesiophobia, have been associated with pain severity and functional impairment in various foot conditions, such as chronic foot pain, plantar heel pain and Achilles tendinopathy [12, 13, 14, 15, 16, 17, 18, 19]. For those with hallux valgus, the impact on health‐related quality of life goes beyond pain and physical function to general health, vitality, social function and mental health, particularly with increasing severity of the deformity [5].

The psychological aspects of foot pain assessed using various measurement tools [20] have also been linked to surgical outcomes for hallux valgus. For example, Klein et al. [21] found no significant association between pre‐operative anxiety/depression scores and post‐operative foot function. In contrast, other studies [22, 23, 24] reported that pre‐operative mental health scores predicted positive post‐operative outcomes. Overall, the findings on the impact of psychological factors on surgical outcomes are inconsistent, which might relate to variability in approaches to assess and categorise pre‐operative mental health status, measures of pain and function, the type of hallux valgus procedure and follow‐up time points. Importantly, it has not been established which psychological factors are associated with pain and function before surgery. Therefore, it is important to establish these relationships using sensitive and validated tools to measure psychological factors, which will enable us to evaluate their impact on surgical outcomes.

The aim of this research is to evaluate the association between various psychological factors and foot pain and function in adults prior to undergoing hallux valgus surgery. These factors which have previously been found to be associated with pain and function in people with foot and ankle pain include emotional factors (e.g. depression, anxiety and stress) and cognitions (e.g. pain catastrophizing and kinesiophobia). By establishing these relationships in the pre‐operative environment, the findings may help inform the design of future investigations of post‐operative outcomes.

## 2 MATERIALS AND METHODS

The ethical approval was obtained from The University of Newcastle's Human Research Ethics Committee (H‐2021‐0343). All participants provided informed consent.

### 2.1 Design

This study was an observational cross‐sectional design conducted over a period of 10 months in 2023, which was chosen to establish baseline associations between psychological factors, pain and function before surgery, which will inform future longitudinal research on their impact on surgical outcomes.

### 2.2 Participants

Participants with hallux valgus, that had consented to having hallux valgus surgery, were recruited from the private practices of three different podiatric surgeons in Sydney, Australia (HO, OA and DL). During the consultation, a podiatric surgical registrar (AK or JJ) who was not directly involved in the patient's care approached the patient and informed them about the research study. To address potential issues related to coercion, several measures were implemented, including ensuring that registrars, rather than the treating surgeons, conducted recruitment; encouraging participants to discuss their involvement with family or friends and emphasising that declining or withdrawing would not impact their care.

To be eligible for inclusion, participants had to meet the following criteria:

1. Aged 18 years or older
2. Able to read basic English
3. A clinical diagnosis of hallux valgus deformity (mild, moderate or severe) as diagnosed by the Manchester Scale [25].
4. Undergoing unilateral and/or bilateral hallux valgus procedures

Participants were excluded from the study if they met any of the following criteria, which were categorised as either related to surgical eligibility or designed to minimise confounding in study outcomes:

#### 2.2.1 Exclusion criteria related to surgical eligibility

1. Not suitable for general anaesthesia (e.g. pregnancy or severe comorbidities)
2. Body mass index (BMI) > 39 kg/m^2^
3. A known active malignancy or ongoing systemic chemotherapy treatment
4. Known opioid dependence
5. Difficulty taking oral medications

#### 2.2.2 Exclusion criteria related to study validity

1. An associated systemic disease (e.g. inflammatory arthritis and diabetes‐related Charcot arthropathy)
2. A previous history of first metatarsal surgery
3. Preference for surgery via minimally invasive techniques (e.g. keyhole surgery)
4. Requirement for additional first metatarsal or digital procedures (e.g. Akin osteotomy)

If the patient met the eligibility criteria and chose to be involved, they were advised to contact the registrars (AK or JJ) via phone or email. In response, AK or JJ provided the patient with the Participant Information Sheet, Consent form and the self‐report outcome measures (in Microsoft Word) via the post or email. The participant was then advised to return the completed forms to AK or JJ via email or the post prior to their planned surgery.

### 2.3 Measures

#### 2.3.1 Dependent variables (primary outcome)

The Manchester–Oxford Foot Questionnaire (MOXFQ) is a 16‐item instrument answered on a five‐point Likert Scale (each item is scored from 0 to 4, with 4 denoting ‘most severe). Raw scale scores were then each converted to a metric from 0 to 100, where 100 denotes the most severe. Function and pain were evaluated with the ‘Walking/standing’ domain (seven items) and foot pain (five items) of the MOXFQ, respectively [26].

#### 2.3.2 Dependent variables (secondary outcome)

Social interaction, as a secondary outcome, was evaluated using the social interaction domain (four items) of the MOXFQ [26].

#### 2.3.3 Independent variables

Age, sex, height, weight, duration of HV symptoms, years of education, previous treatment and HV associated footwear difficulties were self‐reported by participants. BMI was calculated by the researchers (AK or JJ). Additionally, symptoms of depression, anxiety and stress were measured using the Depression, Anxiety and Stress Scale short version (DASS‐21) [27]. Participants needed to report the occurrence of symptoms prior to hallux valgus surgery before rating each item from 0 (‘did not apply to me at all over the last week’) to 3 (‘applied to me very much,’ or ‘most of the time over the past week’). The DASS‐21 has demonstrated reliability, adequate construct validity and robust convergent and discriminant validity [27] and normative data exist for the Australian population for comparison [28].

Kinesiophobia was assessed using the Tampa Scale for Kinesiophobia (TSK), a 17‐item questionnaire designed to measure an individual's fear of movement and/or (re)injury. Participants rated each item on a 4‐point Likert scale from ‘strongly disagree’ (score = 1) to ‘strongly agree’ (score = 4). Scores range from 17 to 68 with higher scores indicating a greater degree of kinesiophobia. Psychometric analysis of the TSK has shown it to have high reliability and internal consistency (Cronbach's *α* = 0.84) [29].

Pain catastrophizing has been described as an overestimation of the threat posed by a person's symptoms and has been defined by Sullivan et al. [30] as ‘an exaggerated negative mental set brought to bear during actual or anticipated pain experience’. It was measured using the Pain Catastrophizing Scale (PCS), a 13‐item questionnaire [31]. Participants were asked to rate the extent to which they experienced each of 13 thoughts or feelings during pain on a 5‐point Likert scale ranging from ‘not at all’ (score = 0) to ‘all the time’ (score = 4). The PCS provides a total score along with three subscale scores that measure rumination, magnification and helplessness. The PCS has demonstrated strong test–re‐test reliability, internal consistency (Cronbach's Alpha = 0.87) and construct validity [31].

### 2.4 Statistical analysis

Descriptive statistics, including mean, median, interquartile range (IQR), standard deviation for continuous variables and percentages for categorical variables, were computed. As psychological continuous predictor variables (depression, anxiety, stress, pain catastrophizing and kinesiophobia) exhibited abnormal distributions (Shapiro–Wilk test and *p* value < 0.05), Spearman’s rank correlations were used to assess potential multi‐collinearity among these variables. Multi‐collinearity occurs when predictor variables are highly correlated, inflating standard errors and hindering the interpretation of independent effects in the model. Identifying highly correlated variables can inform further model selection decisions [32]. Scatter plots confirmed the suitability of linear regression, demonstrating linearity between predictor and dependent variables.

Initially, separate regression analyses were conducted to examine the relationship between each psychological factor and MOXFQ scores while controlling for demographic variables. Moreover, multi‐collinearity in the models was found to be low (VIF <1.5). Including all demographic variables resulted in the model with the lowest Akaike information criterion (AIC), indicating optimal fit. Finally, the final models were constructed to comprehensively analyse each dependent variable, incorporating all the predictor variables. This approach facilitated the identification of a series of variables that significantly impacted each dependent variable while controlling for other variables.

As the constructs of depression, anxiety and stress are interconnected due to common underlying causes, such as genetic predispositions, other vulnerabilities and environmental factors, which can influence all negative emotional states [33], the ‘symptoms of stress’ variable were excluded from the final models due to their high correlation (*r* = 0.78) with depression and their potential impact on multi‐collinearity. Including both variables could mask their specific contributions to the outcomes. Sensitivity analysis was conducted to ensure the robustness of the results and confirm that the exclusion of ‘symptoms of stress' did not compromise the integrity of the findings. Although alternative strategies, such as ridge regression or principal component analysis, could address multi‐collinearity, these methods were not applied in this study to preserve the interpretability of individual predictors [34]. All analyses were conducted at a significance level of *p* < 0.05. Results were presented using coefficients and 95% confidence intervals (CIs). The statistical analyses were carried out using the STATA software (StataCorp. 2023. Stata Statistical Software: Release 18. College Station, TX: StataCorp LLC).

### 2.5 Sample size calculation

A priori sample size calculation was based on a previous study that evaluated the association between pain catastrophizing and first step pain in people with plantar heel pain [12]. Although this study involved a different musculoskeletal condition than the current study focusing on hallux valgus, it provided a relevant effect size estimate (18% variance explained) for the influence of pain catastrophizing on a pain‐related outcome. Using the G*power 3.1.9.7 software [35], it was determined that a minimum of 37 participants would be required to detect a significant association based on a *p* value of 0.05 and power of 0.8 and 10 predictors (i.e. age, sex, BMI, education, duration of pain, depression, anxiety, stress, kinesiophobia and catastrophizing). A total of 41 participants were recruited to account for potential missing data or incomplete surveys.

## 3 RESULTS

The study population demographics (Table 1) revealed a mean age of 53.5 years (SD = 15.4) with most of the *n* = 41 participants identifying as female (78.1%). Participants had a mean BMI of 25.7 kg/m^2^ (SD = 3.8) and an average of 15.3 years of education (SD = 3.3). Pain duration had a median of 60 months (minimum = 3 months, maximum = 360 months and IQR = 72). The median scores for depression, anxiety and stress were 2, 2 and 10, respectively, with IQRs of 8. Median pain catastrophizing and kinesiophobia scores were 12 (IQR = 21) and 33 (IQR = 10), respectively. The mean scores for MOXFQ domains were 44.6 (SD = 30.5) for walking/function, 48.9 (SD = 26.6) for pain and 48.3 (SD = 28.7) for social interactions.

**TABLE 1:** Descriptive statistics of participants' characteristics.

Factors	*N*	Mean	Median	SD	IQR	%
Age (years)	41	53.5	59	15.4	23	‐
Sex						
Male participants	9					21.9
Female participants	32					78.1
BMI	41	25.7	26.1	3.8	5	
Education (years)	35	15.3	16	3.3	5	
Pain duration (months)	38	73.3	60	72.4	72	
Affected limb						
Right	11					26.8
Left	5					12.2
Both	25					61.0
DASS‐21						
Depression	41	5.6	2.0	8.1	8	
Anxiety	41	5.3	2.0	6.6	8	
Stress	41	10.9	10.0	8.9	8	
Pain catastrophizing	41	13.8	12.0	12.9	21	
Kinesiophobia	41	32.5	33.0	8.0	10	
MOXFQ						
Walking/function	41	44.6	42.9	30.5	57.1	
Pain	41	48.9	45.0	26.6	40	
Social interaction	41	48.3	56.3	28.7	43.8	

Abbreviations: %, percentage; DASS‐21, Depression, Anxiety, Stress Scale—short version; IQR, interquartile range; MOXFQ, Manchester—Oxford Foot Questionnaire; SD, standard deviation.

Age showed a moderate negative correlation with walking/function (coefficient −0.40 and *p* = 0.01) and social interactions (coefficient −0.48 and *p* = 0.001). No significant sex differences were observed for any MOXFQ domain (*p* > 0.05). BMI and education were not significantly associated with outcomes, although BMI showed a trend toward significance for the pain domain (coefficient 0.29 and *p* = 0.063). Pain duration was not significantly correlated with any MOXFQ domain (Supporting Information S2).

Spearman’s rank correlation coefficients revealed moderate to strong positive correlations between symptoms of depression with anxiety (0.47, *p* value = 0.002) and stress (0.78, *p* < 0.001). Pain catastrophizing moderately correlated positively with symptoms of depression (0.37, *p* = 0.019), anxiety (0.39, *p* = 0.011) and stress (0.53, *p* < 0.001, as well as with kinesiophobia (0.67, *p* < 0.001) (Supporting Information S1).

Table 2 reveals the relationship between each exposure and MOXFQ outcomes after adjusting for demographics. Symptoms of anxiety and stress were significantly associated with worse foot function (*β* = 1.97, 95% CI 0.96–2.97 and *p* < 0.001 and *β* = 1.45, 95% CI 0.15–2.75 and *p* = 0.031, respectively), more foot pain (*β* = 1.81, 95% CI 0.39–3.22 and *p* = 0.014 and *β* = 1.64, 95% CI 0.39–2.88 and *p* = 0.012, respectively) and poorer social interactions (*β* = 2.02, 95% CI 1.11–2.93 and *p* < 0.001 and *β* = 1.40, 95% CI 0.16–2.64 and *p* = 0.028, respectively). Pain catastrophizing also showed significant associations with worse foot function (*β* = 1.84 and 95% CI 1.38–2.29), more foot pain (*β* = 1.32 and 95% CI 0.70–1.95) and poorer social interactions (*β* = 1.51 and 95% CI 1.13–1.89) (*p* < 0.001). Similarly, kinesiophobia demonstrated significant associations with worse foot function (*β* = 1.89, 95% CI 0.41–3.37 and *p* = 0.014) and poorer social interactions (*β* = 1.61, 95% CI 0.23–2.99 and *p* = 0.024) but not with foot pain. Symptoms of depression did not exhibit significant associations with any MOXFQ domain.

**TABLE 2:** Individual associations (each variable is entered separately with statistically significant demographic variables) between psychological factors and each domain of the MOXFQ (*N* = 41).

Exposures^a^	MOXFQ domains coefficients (95% CI), *p*‐value		
	Walking/function	Pain	Social interactions
DASS‐21 depression	1.09 (−0.22, 2.40), 0.098	1.12 (−0.13, 2.36), 0.077	0.64 (−1.00, 2.28), 0.431
DASS‐21 anxiety	1.97 (0.96, 2.97), <0.001*	1.81 (0.39, 3.22), 0.014*	2.02 (1.11, 2.93), <0.001*
DASS‐21 stress	1.45 (0.15, 2.75), 0.031*	1.64 (0.39, 2.88), 0.012*	1.40 (0.16, 2.64), 0.028*
Pain catastrophizing	1.84 (1.38, 2.29), <0.001*	1.32 (0.70, 1.95), <0.001*	1.51 (1.13, 1.89), <0.001*
Kinesiophobia	1.89 (0.41, 3.37), 0.014*	0.94 (−0.39, 2.26), 0.155	1.61 (0.23, 2.99), 0.024*

Abbreviations: BMI, body mass index; CI, confidence interval; DASS‐21, Depression, Anxiety, Stress Scale—short version; MOXFQ, Manchester–Oxford Foot Questionnaire.

^a^ Controlled for age, sex, education, pain duration and BMI.

*p*‐values <0.05 are considered statistically significant.

**FIGURE 1:**
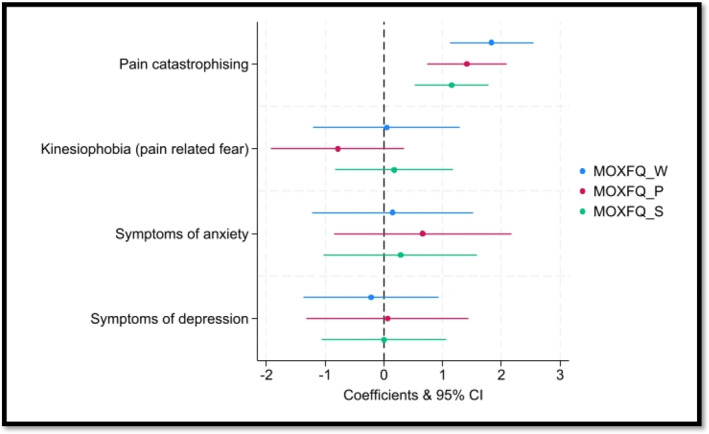
Key predictors of MOXFQ domains (mutually adjusted for each other and demographics). MOXFQ_P, Manchester–Oxford Foot Questionnaire pain domain; MOXFQ_S, Manchester–Oxford Foot Questionnaire social interactions (*N* = 41) and MOXFQ_W, Manchester–Oxford Foot Questionnaire walking domain.

When all the key predictor variables were entered into the regression models simultaneously (Figure 1), only pain catastrophizing emerged as a statistically significant predictor. A one‐unit increase in pain catastrophizing score was associated with a 1.83‐point increase in worse foot function (*β* = 1.83, 95% CI 1.12–2.54 and *p* < 0.001), a 1.41‐point increase in more foot pain (*β* = 1.41, 95% CI 0.73–2.09 and *p* < 0.001) and a 1.15‐point increase in poorer social interaction (*β* = 1.15, 95% CI 0.53–1.78 and *p* = 0.001).

### 3.1 Sensitivity analyses

#### 3.1.1 Inclusion of the ‘symptoms of stress’ variable

An additional sensitivity analysis was conducted to assess interpretability and the impact on other variables with the inclusion of the ‘symptoms of stress' variable in the final models. This was important to ensure that no estimable effects were missed and to verify the robustness of the model. The inclusion of this variable did not change any predictor variable estimates by more than 10%, confirming the stability of the results. Pain catastrophizing remained statistically significant for social interaction (*β* = 1.13 and 95% CI 0.51–1.74), foot function (*β* = 1.79 and 95% CI 1.04–2.54) and foot pain (*β* = 1.31 and 95% CI 0.60–2.01) outcomes. This stability suggests that stress might overlap with other variables, such as depression, potentially explaining similar patterns in data. Despite this overlap, pain catastrophizing demonstrated strong and consistent effects. When stress was included, strong multi‐collinearity was observed. However, the sensitivity analysis confirmed that its exclusion does not result in any missed effects and the model remains stable.

## 4 DISCUSSION

The objective of the study was to evaluate the association between psychological factors with pain and function in adults prior to hallux valgus surgery. Our findings revealed statistically significant associations among various psychological factors and domains of foot pain, function and social interaction, including pain catastrophizing, stress, anxiety and kinesiophobia. In a multivariate regression model, pain catastrophizing emerged as a strong statistically significant predictor of all outcomes when mutually adjusting for depression, anxiety and kinesiophobia but also demographics. Although anxiety and kinesiophobia were not significantly associated with the MOXFQ outcomes in the multivariate model, there were significant associations in univariate analyses. This suggests that anxiety may be associated with pain, function and social interactions and kinesiophobia maybe indirectly linked with pain and social interactions, potentially mediated by pain catastrophizing. These findings highlight the importance of recognising and addressing a range of psychological factors in the pre‐operative environment, even when pain catastrophizing is the most influential predictor.

Our study's findings should be interpreted in the context of limited evidence from foot and ankle studies that have evaluated the association between psychological factors and pain, especially pain catastrophizing. Despite this, our study did observe a significant association between psychological factors, such as anxiety with pain, which aligns with a previous study that investigated a similar relationship in individuals with hallux valgus [21]. Our findings are also similar to a study of 172 adults on a public health wait list for a specialist foot and ankle consultation, where it was revealed that pain catastrophizing explained 14% of the variance in foot pain scores (as measured using the MOXFQ index score) after controlling for age, sex and BMI [18]. These findings align with broader meta‐analyses, which revealed a medium correlation between pain catastrophizing and pain intensity and a medium to large correlation between pain catastrophizing and pain‐related disability in individuals with chronic non‐cancer related pain [36]. Overall, despite the lack of foot and ankle‐specific studies, our results suggest that pain catastrophizing is an important component of patients' experiences and may influence post‐operative outcomes. These findings are consistent with evidence from other surgical contexts, such as spine, hip and knee procedures, where high pain catastrophizing scores have been associated with increased pain, physical disability, opioid use and subsequent use of healthcare services [37].

In our study, when we assessed each predictor variable separately, anxiety, stress, pain catastrophizing and kinesiophobia were all significantly associated with increased foot pain, worse foot function and poorer social interactions. However, when entered in a multivariate model, which demonstrated the simultaneous impact of all exposure variables on MOXFQ outcomes, pain catastrophizing was the only significant predictor of all outcomes. The findings reinforce the concept of psychological models of chronic pain, particularly the fear‐avoidance model, which suggests that individuals with high levels of psychological distress may develop fear‐avoidance beliefs, leading to avoidance behaviours and exacerbation of pain and disability. In this model, a painful injury can trigger catastrophizing thoughts and instil fear, prompting an individual to develop defensive behaviours such as hypervigilance and avoidance of certain movements [38]. Subsequently, this avoidance behaviour may perpetuate further avoidance, dysfunction and potentially symptoms of depression, ultimately exacerbating the experience of pain.

### 4.1 Clinical implications

Understanding the role of psychological factors in the experience of pain in people with hallux valgus has implications for pre‐operative assessments and treatment including education. Our findings highlight the importance of including psychological assessments into the pre‐operative environment to identify individuals who might exhibit catastrophic thoughts about their experience of pain related to their hallux valgus. By recognising and addressing psychological distress early on, specifically related to pain catastrophizing, the surgical team can tailor interventions to target specific concerns and enhance the patient's preparedness for surgery. However, evidence suggests that musculoskeletal clinicians lack the knowledge and skill in evaluating and managing psychological factors related to pain and formal screening for psychosocial factors is not commonly practiced among primary care clinicians, including musculoskeletal specialists [39]. In a cross‐sectional study of patients seen by an orthopaedic surgeon, surgeons were unable to accurately recognise high levels of pain catastrophizing when compared to scores recorded using the PCS [40]. Furthermore, the PCS, which was originally intended for psychologists, is now commonly used by clinicians from various backgrounds without adequate training in mental health [41]. This has had negative consequences for patients with persistent pain as the term has been misused by health professionals to blame patients for their experience of pain and disregard the medical legitimacy of their presentation [42]. Therefore, using psychological tools such as the PCS, without proper training, carries the responsibility to seek appropriate education on their use and interpretation [41]. Fortunately, there is an increasing amount of literature that provides guidance for clinicians specialising in musculoskeletal medicine in the assessment and management of psychological factors as well as supporting the rationale behind their approaches [39, 43]. However, findings of the present study support the need for incorporation of content related to the reasoning and management of psychological factors, such as pain catastrophizing into medical and allied health curriculums. Furthermore, targeted training programmes are required for registered health professionals to competently use psychological tools in practice for patients with musculoskeletal pain [41].

### 4.2 Limitations

Our study should be viewed considering some limitations. First, the cross‐sectional design limits our ability to draw causal inferences and fully explore the complex associations between the psychology of pain and pain outcomes. Second, the study was not designed to evaluate the association between each psychological factor with post‐surgical outcomes. Third, we intended to explore differences by sex, but with only 9 male participants, it would be challenging to conduct a meaningful analysis by sex. Although the overall sample size was determined using a power calculation to detect associations with acceptable power, analysing smaller subgroups may result in underpowered analyses and unreliable results. This necessitates further investigation with a larger more balanced sample size to explore potential sex differences in the impact of these psychological factors on pain and function in adults with hallux valgus. Finally, our study did rely on self‐reported data, which might be influenced by recall bias and the participant's interpretation of the questions. However, we used reliable and validated outcome measures to minimise these effects.

### 4.3 Future research

Future research should not only evaluate the association between pain catastrophizing with pain and function post‐operatively but also evaluate the effectiveness of targeted interventions for pain catastrophizing such as cognitive behavioural therapy, acceptance and commitment therapy and multimodal treatments in the perioperative environment. These approaches, especially cognitive behaviour therapy, have been shown to reduce pain catastrophizing in people with chronic non‐cancer pain. Although the effect sizes are modest, cognitive behavioural therapy may be more likely to be clinically meaningful when targeted to people with high levels of pain catastrophizing [44]. Studies should also evaluate factors that impact pain catastrophizing such as other psychological variables, comorbidities, obesity, smoking and alcohol consumption [45]. In addition, research should explore whether sex differences influence the observed relationships between pain catastrophizing and foot MOXFQ domains. Finally, more research is required to refine pre‐operative screening protocols for the assessment of psychological factors in patients undergoing hallux valgus surgery.

## 5 CONCLUSION

Our study highlights the need for a holistic approach to the management of hallux valgus, one that recognises and addresses the association between psychological and biological health factors. By integrating psychological assessment and intervention into pre‐operative assessments, health professionals can better support individuals with hallux valgus and improve foot health‐related quality of life. More structured education may be required to support health professionals in the assessment of psychological factors, especially pain catastrophizing, in the perioperative environment. Clinicians should consider incorporating validated psychological assessment tools into practice, and researchers should evaluate the effectiveness of targeted psychological interventions to improve surgical outcomes in adults with hallux valgus.

## AUTHOR CONTRIBUTIONS

**Abdel Kak**: Conceptualization; methodology; project administration (lead); writing ‐ original draft; writing—review & editing. **Mehak Batra**: Data curation (lead); formal analysis (lead); writing—original draft; writing—review & editing. **Bircan Erbas**: Formal analysis; writing—original draft; writing—review & editing. **Sean Sadler**: Conceptualization; methodology; writing—original draft; writing—review & editing. **Vivienne Chuter**: Conceptualization; writing—original draft; writing—review & editing. **Jeffery Jenkins**: Conceptualization; investigation; methodology; writing—original draft; writing—review & editing. **Haydar Ozcan**: Conceptualization; writing—review & editing. **Damien Lafferty**: Conceptualization; writing—review & editing. **Ozan Amir**: Conceptualization; writing—review & editing. **Matthew Cotchett**: Conceptualization (lead); methodology (lead); project administration; supervision; writing—original draft; writing—review & editing.

## CONFLICT OF INTEREST STATEMENT

The authors declare that they have no conflicts of interest.

## ETHICS STATEMENT

The ethical approval was obtained from The University of Newcastle's Human Research Ethics Committee (H‐2021‐0343).

## Supporting information

Supporting Information S1

Supporting Information S2

## Data Availability

Data are available upon request from the authors.
