# Supplementary material for: Psychological factors associated with pain and function in adults with hallux valgus
Source: J Foot Ankle Res. 2025 Mar 2;18(1):e70030. doi: 10.1002/jfa2.70030 (PMC11872594; doi:10.1002/jfa2.70030)
Supplement: Supplementary file 1 — Supporting Information S1 [file JFA2-18-e70030-s001.docx]

**Supplementary file 1. Spearman Correlations between psychological predictor variables**

|  | DASS-21 Depression | DASS-21 Anxiety | DASS-21 Stress | Pain catastrophizing | Kinesiophobia |
| --- | --- | --- | --- | --- | --- |
| DASS-21 Depression | 1.00 |  |  |  |  |
| DASS-21 Anxiety | 0.47  0.002* | 1.00 |  |  |  |
| DASS-21 Stress | 0.78*  <0.001 | 0.54  0.000* | 1.00 |  |  |
| Pain catastrophizing | 0.37  0.019* | 0.39  0.011* | 0.53  0.000* | 1.00 |  |
| Kinesiophobia | 0.22  0.158 | 0.33  0.038* | 0.31  0.046* | 0.67  <0.001* | 1.00 |

***DASS-21* Depression, Anxiety, Stress Scale – short version**, ***p-values <0.05 are considered statistically significant**.
