# Supplementary material for: Psychological factors associated with pain and function in adults with hallux valgus
Source: J Foot Ankle Res. 2025 Mar 2;18(1):e70030. doi: 10.1002/jfa2.70030 (PMC11872594; doi:10.1002/jfa2.70030)
Supplement: Supplementary file 2 — Supporting Information S2 [file JFA2-18-e70030-s002.docx]

**Supplementary File 2:** Associations between participants characteristics and MOXFQ Domains

^a^Spearman correlation, ^b^Independent Sample T test, and ^c^Pearson correlation.

| **Factors** | **MOXFQ domains** | | | | | |
| --- | --- | --- | --- | --- | --- | --- |
|  | **Walking/function** | | **Pain** | | **Social Interactions** | |
|  | coefficient or mean (SD) | P value | coefficient or mean (SD) | P value | coefficient or mean (SD) | P value |
| Age | -0.40 | 0.011^a^ | -0.28 | 0.077^a^ | -0.48 | 0.001^a^ |
| Sex  Male, mean (SD) | 42.06 (34.33) | 0.781^b^ | 45.6 (28.8) | 0.670^b^ | 51.38 (21.82) | 0.721^b^ |
| Female, mean (SD) | 5.29 (29.90) |  | 49.8 (26.3) |  | 47.46 (30.57) |  |
| BMI | 0.28 | 0.080^a^ | 0.29 | 0.063^c^ | 0.27 | 0.082^c^ |
| Education (years) | 0.03 | 0.871^a^ | 0.05 | 0.788^a^ | 0.12 | 1.000^c^ |
| Pain duration (months) | 0.05 | 0.783^a^ | 0.03 | 0.837^c^ | 0.11 | 0.506^a^ |
